# Anxiety in Children: The Contribution of Parental Characteristics

**DOI:** 10.3390/children12050553

**Published:** 2025-04-25

**Authors:** Beáta Bécsi, Jenifer Pataki, Gergő József Szőllősi

**Affiliations:** 1 Faculty of Health Sciences, University of Debrecen, 4028 Debrecen, Hungary; becsi.beata@etk.unideb.hu; 2 Department of Integrative Health Sciences, Institute of Health Sciences, Faculty of Health Sciences, University of Debrecen, 4028 Debrecen, Hungary; pataki.jenifer@etk.unideb.hu; 3 Doctoral School of Health Sciences, University of Debrecen, 4032 Debrecen, Hungary; 4 Coordination Center for Research in Social Sciences, Faculty of Economics and Business, University of Debrecen, 4032 Debrecen, Hungary

**Keywords:** anxiety, children, mental health, prevention

## Abstract

**Background/Objectives**: The prevalence of behavioral problems and mental health issues, including anxiety, among children is rising, potentially affecting their long-term well-being and social functioning. Therefore, this study aims to identify the key determinants of children’s health status, with a particular focus on parental mental health, health behaviors, and socio-demographic factors. **Methods**: The data were sourced from a Hungarian representative database from 2019. The analysis was executed using multivariate and multiple logistic regressions. **Results**: Our sample consisted of data from 5603 individuals, of which 775 (14%) completed the children’s module. Significant associations were found between the parent’s tertiary education level (AOR = 3.93 [1.89–8.16]) and the child’s restlessness and anxiety, as well as between the parent’s existing depression and the child’s behavioral difficulties (AOR = 3.22 [1.97–5.28]) and anxiety (AOR = 2.43 [1.37–4.30]). Additionally, a significant association was observed between the parent’s secondary education level (AOR = 3.53 [1.51–8.27]) and the child’s health problems, which was also associated with cases of tertiary education (AOR = 3.17 [1.16–8.69]). **Conclusions**: Our findings suggest that parental education and mental health significantly influence children’s psychological and overall health, which is why targeted prevention and health promotion strategies are essential to support both children and families.

## 1. Introduction

According to the definition made by the World Health Organization (WHO), mental health is not merely the absence of mental disorders, but a far more complex concept, since it simultaneously refers to an individual’s ability to perform well at work, study, or cope with daily stress [1,2]. For children, developing and maintaining activities that support mental well-being is particularly important, as they are among the most emotionally and psychologically vulnerable. This vulnerability mainly arises from the rapid physical and mental changes they might experience, alongside shifts in their social lives, such as their relationships with peers [2]. Notably, many mental health problems that persist into adulthood can often originate from childhood, including common issues such as eating disorders, anxiety, and depression [3].

Globally, approximately 14% of children have experienced some form of mental problem or difficulty, even though the actual prevalence could be relatively higher, since many cases remain unreported and untreated. Among these children, anxiety is one of the most common emotional issues, particularly affecting older children. The global prevalence of anxiety among children aged 10–14 is estimated at 4.4%, increasing to 5.5% among adolescents aged 15–19, according to WHO estimates [2]. Based on data from Europe, Central Asia, and Canada, approximately 33% of adolescents report experiencing anxiety or nervousness multiple times a week, while 29% struggle with sleeping problems [4]. Furthermore, declining trends in life satisfaction, self-rated health, and overall mental well-being have been observed, particularly among girls. This trend is concerning, as early-life mental health issues can persist into adulthood, increasing the risk of long-term psychological disorders [5].

Several factors could contribute to children’s mental well-being, including their positive relationships with peers, emotional regulation, and effective coping strategies. Among these, a supportive family environment is one of the most imperative protective factors for a child’s mental health. However, when this support is lacking, various aspects of family life can become significant risk factors. Inappropriate parenting styles, exposure to abuse or violence, and socioeconomic disadvantages not only contribute to psychological distress, but can also negatively affect a child’s overall well-being, development, and long-term health outcomes [2,6]. Furthermore, negative parenting styles have been associated with an increased risk of developing anxiety and other emotional problems among children [7,8]. Therefore, parental influence, along with family dynamics and structures, play an important role in shaping both the physical and mental health of children [9,10].

Despite the fact that mental health concerns among children could be considered a public health priority, there is a relative lack of large-scale, population-based studies that examine how multiple parental characteristics interact to influence children’s well-being, especially in Hungary. Most existing studies mostly focus on relatively narrow constructs or isolated parental variables, often neglecting broader socioeconomic and health-related factors [11,12,13,14]. Moreover, the research in this domain is frequently limited to respondents with disorders or negative parenting behaviors, and the findings may not be directly transferable to nationwide population levels; therefore, the trends in mental health issues among children cannot be monitored directly [12].

Given this imperative impact and knowledge gap, identifying the specific parental factors which might be associated with children’s mental well-being is essential for both the public health understanding and the development of evidence-based interventions. Accordingly, our study aimed to examine the associations between parental characteristics and the reported presence of anxiety, behavioral difficulties, and health problems in children, using a nationally representative dataset from Hungary.

## 2. Materials and Methods

This cross-sectional study utilized representative data from Hungary’s implementation of the 2019 European Health Interview Survey (EHIS), executed by the Hungarian Central Statistical Office and supervised by EUROSTAT. This survey provides extensive health-related information on adults [15]. It encompasses detailed insights into individual health conditions, medical histories, lifestyles, and socio-demographic attributes. In 2019, for the first time, the Hungarian EHIS survey was extended to include data on children aged 6 months to 14 years. If a selected respondent lived in the same household with one or more children regarding this age group, then they were invited to provide information about one child, chosen based on a predefined selection protocol. Furthermore, of the 5603 individuals who completed the adult questionnaire, 775 (13.83%) provided responses regarding a child living in the household. Consequently, all information concerning the children’s health and well-being in this study reflects the parental reports and perceptions, which means that the analysis was based on data from the individuals who provided responses on behalf of their children. The 2019 dataset was selected for analysis, as it is the most recent, publicly available, and methodologically complete wave of EHIS data in Hungary. Importantly, it is also the only wave to include a child health module, making it uniquely suitable for examining the child-level outcomes in a nationally representative sample. The data collection was conducted between September and December 2019; participants could complete the questionnaire either independently online or with the assistance of trained interviewers via face-to-face interviews, using tablet-based survey tools. The sampling procedure ensured national representativeness across Hungary, with the respondents being randomly selected from the national address registry across 510 municipalities using stratified sampling and a standardized methodology, as defined by the EHIS protocols.

The database included information about the respondents’ socio-demographic characteristics, such as education level (primary/secondary/tertiary), perceived income (good/bad), type of residence (urban/rural), and marital status (partnered, married/single). In the Hungarian educational system, “primary” refers to the completion of general schooling up to the 8th grade, typically by the age of 14; “secondary” refers to general, high school, or vocational training, mostly up to the age of 18; and “tertiary” denotes higher education, including college or university degrees. Data were collected on parental health behaviors such as smoking (smoking/not smoking), physical activity (inactive/moderately active/active), self-perceived health status (good/poor), how much the respondent believes they can do for their health (much/little), and the most recent visit to a doctor and specialist (within 1 year/more than 1 year). Additionally, we considered whether the parent suffered from (self-identified) depression (yes/no) as a variable. For children of the participating parents, the following outcomes were examined in the study: the presence of a health problem (yes/no), restlessness, irritability, or anxiety (yes/no), and behavioral difficulties (yes/no). These items were taken directly from the original EHIS child module and used without recoding, except for the dichotomization of responses where applicable. The anxiety-related item referred to whether the child appeared irritable, restless, or anxious at least once a month or not. All child-related questions referred to the child’s current status as perceived by the parent, rather than to a defined retrospective period. Behavioral difficulties were assessed by asking whether the child had problems with conduct, overactivity, or concentration at least once a month. Health problems included any chronic or long-term physical or mental condition identified by the parent. All child-related data were provided by one selected parent, based on the predefined EHIS sampling protocols.

Pearson’s chi-square tests were used to determine the proportional differences between the issues examined affecting the children and the presence of the various parental factors. To identify factors associated with the children’s mental and health well-being, confounder-adjusted multivariate and multiple logistic regression models were performed using the enter method by including all relevant variables simultaneously in the models. The findings were represented through adjusted odds ratios and 95% confidence intervals. The statistical analysis utilized Stata Statistical Software (version 13.0, Stata Corp, College Station, TX, USA), with the significance set at *p* < 0.05.

## 3. Results

Based on the gender distribution of the children, 48.26% were girls and 51.74% were boys. When considering the parents’ examined characteristics, higher proportions had a secondary level of education (52.77%), belonged to the lower-income category (60.63%), and were married or in a partnership (90.84%). Additionally, 16.96% of the parents reported experiencing depression during the study. According to the survey, 59.35% of the children were anxious, restless, or irritable, 13.04% had some type of health problem, and 17.44% had behavioral difficulties.

### 3.1. Results of Chi-Square Tests

#### 3.1.1. Children’s Health Problems

A significant difference was found in the prevalence of health problems based on several parental characteristics. The children of single parents experienced more health problems (23%) than those of married parents (12%) (*p* = 0.019) (Table 1). Parental education was also associated with this outcome (*p* = 0.007), with the highest prevalence reported by the parents with secondary education (17%), followed by those with tertiary (10%) and primary educations (6%). Income was strongly related to reported child health problems: the parents from lower-income households reported more health problems (16%) compared to those from higher-income households (8%) (*p* < 0.001). In the case of the type of residence, there was no significant difference (*p* = 0.768) between the parents from urban and rural areas regarding the children’s health problems. No significant associations were found between self-perceived health status (*p* = 0.794), parental depression (*p* = 0.443), how much someone can do for their health (*p* = 0.491), or smoking status (*p* = 0.709) and child health problems. Likewise, the timing of the last GP visit (*p* = 0.567) or specialist visit (*p* = 0.757) and physical activity (*p* = 0.813) were not associated with this outcome.

#### 3.1.2. Children’s Anxiety

Parental education was significantly associated with children’s anxiety (*p* < 0.001). The parents with tertiary education reported the highest prevalence (72%), followed by those with secondary (55%) and primary educations (51%). Parental depression was another strong factor: the parents who reported depression observed anxiety in their children more frequently (74%) than those without depression (56%) (*p* = 0.002). The residence was also associated: the parents from urban areas reported more anxiety (63%) than the ones from rural areas (53%) (*p* = 0.023). However, no significant associations were found for marital status (*p* = 0.740), income (*p* = 0.138), self-perceived health status (*p* = 0.142), how much someone can do for their health (*p* = 0.390), smoking status (*p* = 0.889), or physical activity (*p* = 0.203). Neither the timing of GP visits (*p* = 0.345) nor that of specialist visits (*p* = 0.966) were significantly associated with anxiety.

#### 3.1.3. Children’s Behavioral Difficulties

The parents with primary education reported more behavioral problems in their children (26%) than those with secondary (16%) or tertiary educations (16%) (*p* = 0.047). Parental depression was a particularly strong factor (*p* < 0.001): the children of parents with depression had a higher prevalence of behavioral difficulties (34%) than those without (14%). Self-perceived health status (poor = 38% vs. good = 17%) was also associated with this outcome (*p* = 0.011), as was the factor of how much someone can do for their health (much = 65% vs. little = 26%) (*p* = 0.030). A significant difference was found in relation to the time since the last specialist visit: the children whose parents had not seen a specialist in over a year had more behavioral problems (21%) compared to those who had had more recent contact (15%) (*p* = 0.042). No significant differences were found with respect to marital status (*p* = 0.531), income (*p* = 0.331), type of residence (*p* = 0.724), smoking status (*p* = 0.261), physical activity (*p* = 0.864), or recent GP visits (*p* = 0.619).

### 3.2. Results of Logistic Regression Models

#### 3.2.1. Children’s Health Problems

**Figure 1:**
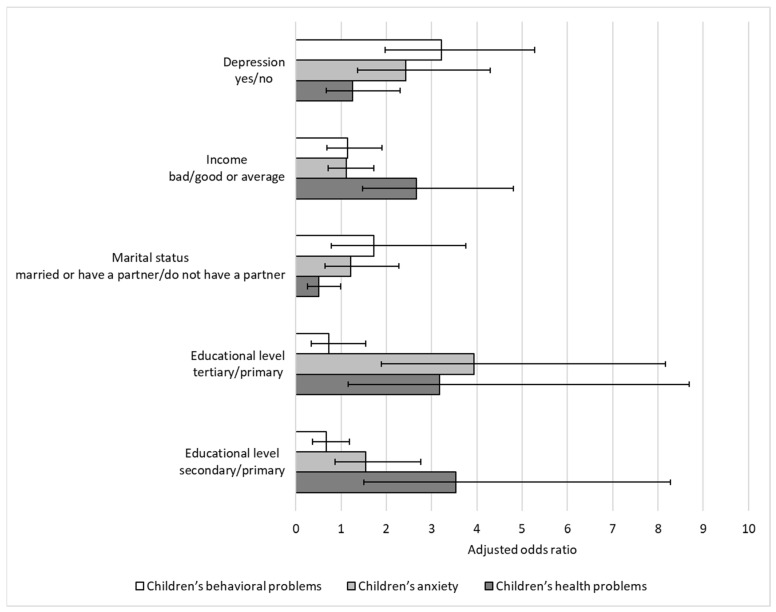
Adjusted odds ratios (AORs) and 95% confidence intervals (CIs) for factors associated with children’s health problems, anxiety, and behavioral problems based on confounder-adjusted multiple and multivariate logistic regression models. All models are adjusted for type of residence, self-perceived health status, how much an individual can do for their own health, smoking status, last meeting with doctor or specialist, and physical activity.

Based on the confounder-adjusted multiple logistic regression models, we found that compared to the children of parents with primary education, the children of parents with secondary education had 3.53 (AOR = 3.53 [1.51–8.28]) times higher odds of reporting a child’s health problem (Figure 1). The parents with tertiary education had a slightly lower odds ratio (AOR = 3.17 [1.16–8.69]), but this effect remains statistically significant. The children of married parents had a lower likelihood of reporting any health issues, compared to the children of single parents (AOR = 0.51 [0.26–0.99]). Higher incomes were associated with increased odds of reporting health problems (AOR = 2.67 [1.48–4.79]). Parental depression appeared to have no significant impact on the odds of reporting a child’s health problem (AOR = 1.25 [0.67–2.31]). No significant association was found between the type of residence (AOR = 1.21 [0.73–1.99]), the self-perceived health status (AOR = 1.08 [0.28–4.16]), or the factor of how much someone can do for their own health (AOR = 1.29 [0.64–2.62]), and the reporting of children’s health problems. Parental smoking habits (AOR = 1.04 [0.62–1.74]), as well as visits to general practitioners (AOR = 0.94 [0.54–1.64]) and specialists (AOR = 1.03 [0.62–1.71]) within one year, did not prove to be significant factors. Similarly, there was no significant correlation between the parent’s physical activity level (moderately active AOR = 1.23 [0.67–2.25]; active AOR = 1.25 [0.59–2.61]) and the child’s health issues.

#### 3.2.2. Children’s Anxiety

The parents with secondary education levels were 54.7% (AOR = 1.55 [0.87–2.76]) more likely to report restlessness or irritability in their children compared to the parents with primary education. However, this result was not statistically significant. The tertiary education level of a parent significantly increased the odds of reporting restlessness or irritability by nearly four times (AOR = 3.93 [1.89–8.16]) compared to a primary education level. Family structure did not appear to influence the reporting of anxiety in children (AOR = 1.21 [0.64–2.28]). Higher income (AOR = 1.12 [0.72–1.73]) and type of residence (AOR = 1.35 [0.90–2.03]) were not significantly associated with restlessness or irritability in children. Parental depression seemed to be a significant predictor. The parents experiencing depression were 2.43 times (AOR = 2.43 [1.37–4.30]) more likely to report restlessness or irritability in their children. No significant association was found between the parent’s type of residence and the child’s anxiety (AOR = 1.35 [0.90–2.03]. The poor self-perceived health of a parent increased the odds of reporting restlessness or irritability, but the result was not statistically significant (AOR = 2.76 [0.79–9.60]). Behavioral factors such as smoking (AOR = 0.72 [0.48–1.10]), physical activity level (moderately active AOR = 0.74 [0.45–1.20]; active AOR = 0.68 [0.37–1.23]), or visits to general practitioners (AOR = 0.68 [0.44–1.06]) and specialists (AOR = 1.42 [0.93–2.16]) within one year did not seem to impact restlessness or irritability in the model.

#### 3.2.3. Children’s Behavioral Difficulties

Educational attainment did not show a significant association with the likelihood of reporting behavioral difficulties. Neither for the children of parents with a secondary education (AOR = 0.67 [0.37–1.18]), nor for those with tertiary education (AOR = 0.73 [0.34–1.55]), were the associations significant, although in both cases, the likelihood of behavioral difficulties was relatively lower compared with that of primary education. Family structure (AOR = 1.72 [0.78–3.76]), household income (AOR = 1.14 [0.69–1.90]), residence type (AOR = 1.18 [0.75–1.87]), and the factor of how much someone can do for their health (AOR = 1.29 [0.72–2.34]) did not appear to play a major role in predicting behavioral difficulties. Depression was a strong and statistically significant predictor, as the parents with depression were three times more likely to report behavioral difficulties (AOR = 3.22 [1.97–5.28]). The parents perceiving their own health negatively had 2.1 times higher odds of reporting that their children had behavioral difficulties, but the effect was not statistically significant (AOR = 2.10 [0.78–5.66]). Similarly, there was no significant associations between parental smoking habits (AOR = 0.92 [0.58–1.46]), physical activity levels (moderately active AOR = 0.97 [0.58–1.63]; active AOR = 1.11 [0.57–2.15]), or visits to general practitioners (AOR = 0.99 [0.61–1.61]) and behavioral difficulties in children. However, if a parent had not visited a specialist in over a year, it significantly increased the odds of behavioral difficulties in their child (AOR = 1.77 [1.12–2.80]).

## 4. Discussion

Identifying the factors that might contribute to childhood mental and health problems is crucial for targeted intervention and prevention, as negative changes during this life stage can affect the later adult quality of life [16,17,18]. Since the early detection of such problems often relies on caregiver observations, it is important to note that, in this study, the data regarding children’s health and psychological status were collected exclusively through parental reports. So, a methodological consideration is that all data regarding the children’s health and psychological status were provided by the parents, which might also introduce the possibility of reporting bias, as no standardized screening tools or clinical diagnoses were applied to confirm the presence of health or behavioral conditions. Therefore, the variability in parents’ perceptions, awareness, and willingness to report sensitive information may also influence the results to some extent. However, the importance of our research results lies in the extensive and representative database we had, with numerous variables that simultaneously contained relevant data on both the parent and child. This contributed to the identification of several parental factors related to the mental and physical well-being of children in Hungary.

The findings indicated that the children of parents with lower incomes were more likely to be reported as having health-related problems, a result confirmed by other studies [19,20]. This association may reflect the impact of contextual factors such as limited access to healthcare services, poorer living conditions, and increased exposure to environmental and psychosocial stressors. Although we did not find a significant association between the family’s financial status and the child’s anxiety or behavioral problems, other research suggests that children growing up in lower-income households are more prone to psychosomatic issues [20].

While our model showed that the children whose parents had secondary or tertiary education levels had a higher likelihood of suffering from health problems, and that the children of higher-educated parents were more prone to anxiety, irritability, and restlessness, these paradoxical findings may be influenced by multiple factors, including differentials in parental awareness and perceptions of child health issues, increased academic and social pressures on the children, or differences in parenting styles and expectations that contribute to stress and emotional challenges, which were not measured in our study. However, some results have confirmed the association between parental education levels and children’s mental well-being, but these studies found negative correlations among children whose parents had lower education levels concerning perceived health and mental problems [21,22]. This is attributed to more challenging access to the healthcare system linked to a lower socioeconomic status, as well as a higher number of negative life events, which have both mental and biological consequences. Additionally, individuals with lower socioeconomic status are less committed to a healthy lifestyle compared to their higher-status peers [19,22]. Our results may be biased, since the children’s mental and health problems were categorized and reported based on parental self-reports, which could introduce reporting bias and affect the accuracy of the findings, since higher-educated parents may be more likely to notice problems in their children than parents with less knowledge in the field of health promotion [23]. Moreover, it is important to note that a parent’s own psychological state, such as suffering from depression, may influence how they perceive and report their child’s behavior or emotional well-being. For instance, depressive symptoms may lead to a more negative interpretation of the child’s conduct, or in some cases, underreporting due to reduced attentiveness or emotional withdrawal [24,25]. Likewise, higher levels of education may correspond to an increased awareness of developmental norms or psychological symptoms, making these parents more likely to recognize and report subtle difficulties [26]. These differences in interpretation and reporting practices may partly explain some of the associations observed and highlight the importance of cautious interpretation when relying exclusively on single-informant, self-reported data.

Our findings also showed that married or partnered parents proved to be a protective factor, with a lower likelihood of health problems in the children of married/partnered parents. This can be explained by the fact that single-parent families are more likely to have lower incomes, which might be associated with a lower socioeconomic background, which has a negative correlation with both child development and health status [27,28].

Parental depression was significantly associated with children’s health outcomes in terms of increased restlessness, irritability, and behavioral difficulties in children, suggesting a potentially imperative association. This relationship may be attributed to several inter-related factors. Hence, parental depression may be associated with reduced emotional availability, altered parenting practices, and potentially stressful family dynamics, contributing to a less supportive home environment. Additionally, children whose parents have depression may experience higher levels of stress, which can manifest as emotional and behavioral challenges. The parent’s condition can also indirectly affect the child’s well-being by influencing academic performance, health behaviors, and social interactions, further exacerbating psychological distress [29,30,31].

These findings highlight the complex association between parental factors and children’s mental and physical well-being, highlighting the need for targeted interventions. Future research should explore additional mediating factors, such as parenting styles, stress management, and access to healthcare, to better understand these relationships. Strengthening prevention strategies and family support programs could play a crucial role in mitigating the negative impact of parental factors on children’s health outcomes.

### Strengths and Weaknesses

This research utilized datasets from the EHIS, providing a representative sample of Hungary’s adult population. Furthermore, as the analysis was based on data from the Hungarian population, the generalizability of the findings to other countries may be limited due to differences in the cultural, economic, and healthcare system contexts. However, it is important to note that the questionnaires were self-reported, which could lead to a potential under-representation of the results. This may be due to the parents’ fears of stigmatization, leading them to provide less accurate responses regarding their own health, socioeconomic status, and their children’s negative physical and mental conditions. Moreover, since the data regarding the children were derived exclusively from parental reporting and were not validated through clinical assessments or standardized screening tools, some degree of reporting bias or misclassification may also be present in this study. In addition, due to the cross-sectional nature of the data, causal relationships cannot be established; therefore, all findings should be interpreted as associations, rather than directional effects. Furthermore, the available variables were limited by the original dataset, which may have prevented the inclusion of certain potentially relevant confounders. Nevertheless, our research was able to identify several factors supported by other works in the literature, highlighting the relevance and consistency of our findings. This might raise the possibility of omitted variable bias, which should be considered during the interpretation of the results obtained. Future studies could benefit from the integration of screening-validated sources to improve the accuracy and depth of the child health assessments, and to explore additional factors that might serve as targets for prevention and intervention.

## 5. Conclusions

In conclusion, this study highlights the complex relationships of parental and socioeconomic factors in shaping children’s mental and physical health. Lower parental incomes, single-parent households, and parental depression were strongly associated with adverse outcomes reported in children, while parental education presented nuanced results that require further investigation. The findings underscore the importance of early interventions targeting at-risk families, as well as the need for comprehensive support systems to mitigate the long-term effects of these factors on children’s well-being.

## Figures and Tables

**Table 1:** Associations between socioeconomic and health-related factors of respondents in relation with their children’s health, anxiety, and behavioral problems. Significant findings are marked with “*”.

Parent-Related Factors		Children’s Health Problems			Children’s Anxiety			Children’s Behavioral Problems		
		Yes (%)	No (%)	*p*-Value	Yes (%)	No (%)	*p*-Value	Yes (%)	No (%)	*p*-Value
Educational level	primary	7 (6%)	104 (94%)		40 (51%)	39 (49%)		28 (26%)	81 (74%)	
	secondary	61 (17%)	305 (83%)	0.007 *	155 (55%)	129 (45%)	<0.001 *	58 (16%)	308 (84%)	0.047 *
	tertiary	22 (10%)	191 (90%)		116 (72%)	45 (28%)		34 (16%)	179 (84%)	
Marital status	married or have a partner	76 (12%)	544 (88%)	0.019 *	275 (59%)	189 (41%)	0.740	111 (18%)	507 (82%)	0.531
	do not have a partner	14 (23%)	47 (77%)		29 (56%)	22 (44%)		9 (15%)	52 (85%)	
Income	bad	70 (16%)	358 (84%)	<0.001 *	183 (57%)	139 (43%)	0.138	79 (19%)	347 (81%)	0.331
	good/average	20 (8%)	242 (92%)		128 (63%)	74 (37%)		41 (16%)	221 (84%)	
Type of residence	rural	32 (13%)	223 (87%)	0.768	104 (53%)	92 (47%)	0.023 *	46 (18%)	208 (82%)	0.724
	urban	58 (13%)	f377 (87%)		207 (63%)	121 (37%)		74 (17%)	360 (83%)	
Self-perceived health status	good	87 (13%)	583 (87%)	0.794	297 (59%)	209 (41%)	0.142	112 (17%)	554 (83%)	0.011 *
	poor	3 (15%)	17 (85%)		13 (76%)	4 (24%)		8 (38%)	13 (62%)	
Depression	yes	17 (15%)	94 (85%)	0.443	63 (74%)	22 (26%)	0.002 *	38 (34%)	74 (66%)	<0.001 *
	no	72 (13%)	498 (87%)		242 (56%)	189 (44%)		81 (14%)	486 (86%)	
How much can you do for your health?	much	77 (13%)	526 (87%)	0.491	267 (58%)	190 (42%)	0.390	98 (65%)	52 (35%)	0.030 *
	little	13 (15%)	71 (85%)		41 (64%)	23 (36%)		22 (26%)	63 (74%)	
Smoking status	yes	29 (12%)	205 (88%)	0.709	111 (60%)	75 (40%)	0.889	46 (20%)	187 (80%)	0.261
	no	61 (13%)	394 (87%)		199 (59%)	138 (41%)		74 (16%)	380 (84%)	
Last meeting with GP	<12 months	65 (14%)	412 (86%)	0.567	215 (61%)	139 (39%)	0.345	81 (17%)	393 (83%)	0.619
	≥12 months	25 (12%)	183 (88%)		93 (56%)	72 (44%)		39 (19%)	170 (81%)	
Last meeting with specialist	<12 months	54 (14%)	345 (86%)	0.757	181 (59%)	124 (41%)	0.966	59 (15%)	338 (85%)	0.042 *
	≥12 months	36 (13%)	247 (87%)		126 (59%)	87 (41%)		59 (21%)	224 (79%)	
Physical activity	inactive	18 (12%)	131 (88%)	0.813	75 (66%)	38 (34%)	0.203	28 (19%)	120 (81%)	0.864
	moderately active	53 (13%)	358 (87%)		179 (58%)	129 (42%)		70 (17%)	339 (83%)	
	active	19 (15%)	111 (85%)		57 (55%)	46 (45%)		22 (17%)	109 (83%)	

## Data Availability

The data underlying this study’s findings are sourced from the Hungarian Central Statistical Office and are subject to access restrictions. They were used under license for this study and are not publicly accessible. However, they can be obtained from the corresponding author upon reasonable request and with permission from the Hungarian Central Statistical Office.
